# A portable on-axis laser-heating system for near-90° X-ray spectroscopy: application to ferropericlase and iron silicide

**DOI:** 10.1107/S1600577519017041

**Published:** 2020-02-13

**Authors:** Georg Spiekermann, Ilya Kupenko, Sylvain Petitgirard, Manuel Harder, Alexander Nyrow, Christopher Weis, Christian Albers, Nicole Biedermann, Lélia Libon, Christoph J. Sahle, Valerio Cerantola, Konstantin Glazyrin, Zuzana Konôpková, Ryosuke Sinmyo, Wolfgang Morgenroth, Ilya Sergueev, Hasan Yavaş, Leonid Dubrovinsky, Metin Tolan, Christian Sternemann, Max Wilke

**Affiliations:** a Insitute of Geosciences, University of Potsdam, Karl-Liebknecht-Straße 24-25, 14476 Potsdam, Germany; b Deutsches Elektronen-Synchrotron (DESY), Photon Science, 22607 Hamburg, Germany; c GFZ German Research Centre for Geosciences, 14473 Potsdam, Germany; d Institut für Mineralogie, Universität Münster, 48149 Münster, Germany; e Institut für Geochemie und Petrologie, ETH Zürich, 8092 Zürich, Switzerland; f Fakultät Physik/DELTA, Technische Universität Dortmund, 44227 Dortmund, Germany; g European XFEL, 22869 Schenefeld, Germany; h ESRF – The European Synchrotron, 38000 Grenoble, France; i School of Science and Technology, Meiji University, Kanagawa, Japan; j Institut für Geowissenschaften, Universität Frankfurt, 60438 Frankfurt am Main, Germany; k Coherent Light Source, SLAC National Accelerator Laboratory, 2575 Sand Hill Road, Menlo Park, CA 94025, USA; l Bayerisches Geoinstitut (BGI), 95440 Bayreuth, Germany

**Keywords:** laser heating, extreme conditions, X-ray emission spectroscopy, high pressure, nuclear resonant inelastic scattering

## Abstract

A portable IR fiber laser-heating system, optimized for X-ray emission spectroscopy (XES) and nuclear inelastic scattering (NIS) spectroscopy with signal collection through the radial opening of diamond anvil cells near 90°with respect to the incident X-ray beam, is presented. The system offers double-sided on-axis heating by a single laser source and zero attenuation of incoming X-rays other than by the high-pressure environment. A description of the system, which has been tested for pressures above 100 GPa and temperatures up to 3000 K, is given. The XES spectra of laser-heated Mg_0.67_Fe_0.33_O demonstrate the potential to map the iron spin state in the pressure–temperature range of the Earth’s lower mantle, and the NIS spectra of laser-heated FeSi give access to the sound velocity of this candidate of a phase inside the Earth’s core. This portable system represents one of the few bridges across the gap between laser heating and high-resolution X-ray spectroscopies with signal collection near 90°.

## Abstract 2

A portable laser-heating system, optimized for synchrotron X-ray spectroscopy, is presented.

## 1. Introduction   

**Figure 1:**
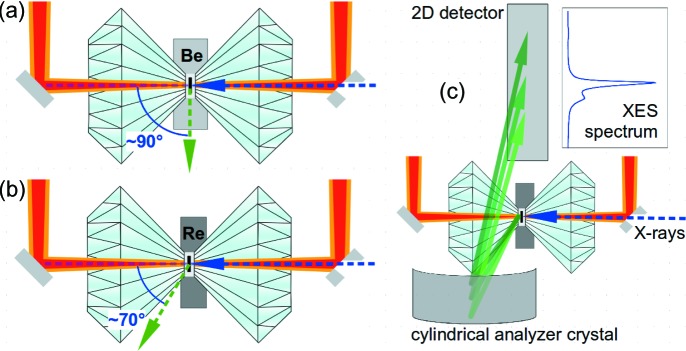
Sketches of the experimental laser-heated DAC setups with on-axis heating and observation (red and orange paths, respectively), collinear X-ray incidence (dashed blue arrows) and salient X-ray signal (green arrows) at ∼90° scattering angle through a Be gasket and at ∼70° (*i.e.* near 90°) through the downstream diamond. (*a*) Setup with collection at ∼90° through an X-ray transparent Be gasket, as used here for NIS measurements. (*b*) Setup with collection at ∼70°, passing a rhenium gasket through the downstream diamond. The indentation wall on the downstream side of the gasket was ground off. (*c*) Sketch to illustrate the arrangement of the DAC, analyzer crystal and detector in the case of an energy-dispersive von Hámos spectrometer.

The experimental investigation of structural properties, phase relations, chemical equilibria and transport properties of material of the deep Earth requires pressures and temperatures that are statically achievable only by the combination of diamond anvil cells (DACs) and laser heating (LH) (Ming & Bassett, 1974 ▸, 2001 ▸; Boehler & Chopelas, 1991 ▸; Lin *et al.*, 2005 ▸; Prakapenka *et al.*, 2008 ▸; Meng *et al.*, 2015 ▸). In a conventional DAC, the sample and a pressure medium are enclosed between two diamond anvils and a metal gasket and compressed by a force applied on the two precisely aligned anvils (Fig. 1 ▸). Samples can be investigated *in situ* at high pressure and temperature with various X-ray spectroscopic techniques, X-ray diffraction (XRD) and optical spectroscopy (Lin *et al.*, 2004 ▸, 2005 ▸; Zhao *et al.*, 2004 ▸; Murakami *et al.*, 2009 ▸; Meng *et al.*, 2015 ▸; Prescher *et al.*, 2015 ▸; Liu *et al.*, 2019 ▸).

Recent years have witnessed an increase of interest in the application of X-ray emission spectroscopy (XES) and nuclear inelastic scattering (NIS) to samples inside a DAC, as they allow for unique insight into matter at extreme pressure (Lin *et al.*, 2007 ▸; Murphy *et al.*, 2013 ▸; Prescher *et al.*, 2015 ▸; Cerantola *et al.*, 2017 ▸; Liu *et al.*, 2018 ▸; Weis *et al.*, 2017 ▸; Spiekermann *et al.*, 2019 ▸). XES is a powerful tool for determining the local structure and electronic spin configuration (Badro *et al.*, 1999 ▸; Lin *et al.*, 2005 ▸, 2007 ▸; Glatzel *et al.*, 2009 ▸; Spiekermann *et al.*, 2019 ▸). NIS yields the phonon density of states of Mössbauer isotopes such as ^57^Fe and provides, among other parameters, the mean sound velocity [Debye velocity, *V*_D_, see *e.g.* Chumakov *et al.* (2009 ▸)], which together with density and bulk modulus are the necessary parameters for calculating the compressional and shear wave sound velocities of materials at extreme pressures (Prescher *et al.*, 2015 ▸; Kupenko *et al.*, 2015 ▸).

The experimental setups for XES and NIS occupy a special position in the wide range of synchrotron X-ray measurement techniques, as the signal is preferably collected in a window of about −25° to +25° around a 90° scattering angle. For XES, the reason is to reduce Thompson scattering, making use of the polarization plane of the source (Glatzel & Bergmann, 2005 ▸), and to exploit the DAC itself for reducing scattering background. For NIS of a sample in the DAC, the main reason is the radial opening of panoramic DACs, which allows for coverage of a significantly larger solid angle when loaded with X-ray transparent beryllium gaskets, as avalanche photo-diodes (APDs) can be placed at about 3 mm from the sample (Kupenko *et al.*, 2015 ▸). Both XES and NIS benefit from the fact that in measurements near a 90° scattering angle the signal is attenuated by only about ∼400 µm of diamond or between 1 mm and 3 mm of Be gasket, as opposed to between 1.3 mm and 2.3 mm in the forward scattering direction, if it is impossible to use miniature diamonds (Petitgirard *et al.*, 2017 ▸) or perforated diamonds.

Two near-90° measurement geometries for XES and NIS are shown in Fig. 1 ▸. In a typical synchrotron experiment setup, the X-ray beam enters the DAC axially through the upstream diamond (right-hand-side diamonds in Fig. 1 ▸). If the DAC is loaded with a beryllium gasket, the emitted XES or NIS signal is collected at ∼90° with respect to the incident beam [Fig. 1 ▸(*a*)] (Chumakov & Sturhahn, 1999 ▸; Mao *et al.*, 2001 ▸; Lin *et al.*, 2005 ▸, 2007 ▸; Murphy *et al.*, 2013 ▸; Kupenko *et al.*, 2015 ▸; Prescher *et al.*, 2015 ▸). When the DAC is loaded with a rhenium gasket, the XES signal passes through about 300–500 µm of diamond and can be collected at *ca* 70° [Fig. 1 ▸(*b*) ▸] when the downstream indentation wall of the gasket has been ground off before loading [indicated by the different penetration depths of the diamonds in the gasket in Fig. 1(*b*)]. Although this differs by 20° from 90°, we refer to this arrangement as near 90°, because both setups pose very similar requirements to the DAC, the LH system and the positioning of detectors. In the case of XES measurements with a von Hámos spectrometer, a fraction of this salient emission radiation falls on one or several cylindrically bent crystal analyzers and is dispersed by them onto a 2D detector [Fig. 1(*c*)] (von Hámos, 1932 ▸; Alonso-Mori *et al.*, 2012 ▸; Szlachetko *et al.*, 2012 ▸; Weis *et al.*, 2019 ▸). DACs with customized wider radial openings, such as panoramic DACs (Mao *et al.*, 2001 ▸), are essential for such experiments.

The application of XES or NIS measurements with laser heating, despite its great potential for investigation of samples under *in situ* conditions of the Earth’s mantle, remains limited to few groups and beamlines (Zhao *et al.*, 2004 ▸; Lin *et al.*, 2007 ▸; Rueff *et al.*, 2008 ▸; Dubrovinsky *et al.*, 2009 ▸; Murphy *et al.*, 2013 ▸; Kupenko *et al.*, 2015 ▸; Aprilis *et al.*, 2017 ▸; Weis *et al.*, 2019 ▸).

One of the reasons for this limitation lies in the fact that commonly only dedicated X-ray spectroscopy beamlines provide the requirements for a routine combination of laser heating and NIS or XES. For NIS, the requirements are a scannable beam with meV-resolution at 14.4 keV, and a suitable bunch-timing regime. The flux in this setting should be of the order of 10^9^ photons s^−1^. The permanent installation of an LH system at a beamline capable of NIS is very rare (Zhao *et al.*, 2004 ▸), and thus the majority of laser-heated NIS measurements have been carried out with portable LH systems (Dubrovinsky *et al.*, 2009 ▸; Kupenko *et al.*, 2015 ▸; Aprilis *et al.*, 2017 ▸).

The beamline requirements for XES with LH-DAC consist of a high-flux beam of 10^13^ photons s^−1^ or more between 10 keV and 15 keV, preferably monochromated with eV-resolution and energy-scannable with constant focus. These requirements are rarely met by beamlines where most LH systems are installed. This may explain why the two studies reporting laser-heated XES at XRD beamlines (Lin *et al.*, 2007 ▸; Rueff *et al.*, 2008 ▸), despite the potential of this combination, were not followed by similar studies.

Small and optimized LH systems, portable to X-ray spectroscopy beamlines, are the answer to overcome this gap. To the best of our knowledge, the present manuscript describes the first application of XES to a laser-heating experiment at a dedicated X-ray spectroscopy beamline, and the first application of a portable LH system applied to NIS outside the European Synchrotron Radiation Facility (ESRF).

We present a portable, versatile near-IR LH system, which is optimized for X-ray spectroscopy applications such as XES and NIS at or near 90°. Despite this optimization and portability, the system shares several advantages of common stationary LH systems installed at extreme conditions beamlines (Prakapenka *et al.*, 2008 ▸; Meng *et al.*, 2015 ▸; Anzellini *et al.*, 2018 ▸; Smith *et al.*, 2018 ▸): double-sided on-axis heating with specialized refractive objectives, double-sided radiospectrometric temperature measurement, constant control of the probed sample spot and full motorized control of all degrees of freedom. Zero attenuation of the incident X-rays is achieved by a pinhole in the upstream mirror. In the following sections, we describe the concept and details of this LH system and show test-case XES and NIS measurements of Mg_0.67_Fe_0.33_O and FeSi, respectively, carried out at beamline P01 at PETRA III, DESY.

## 2. Design and elements of the system   

### 2.1. General concept   

**Figure 2:**
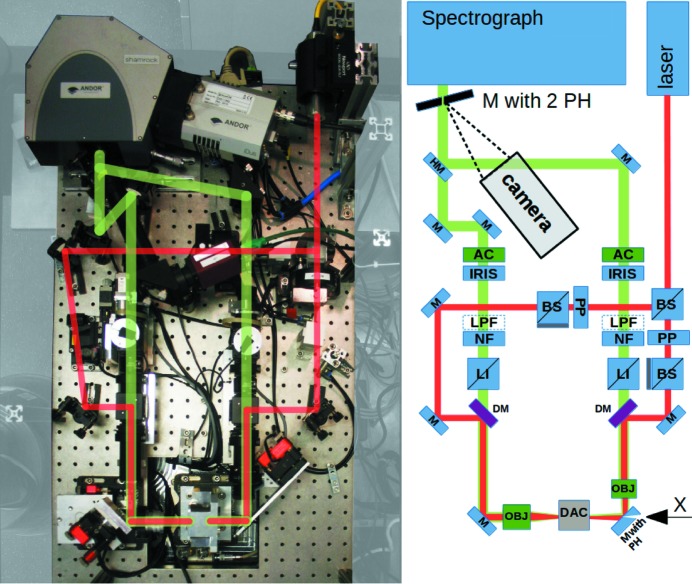
Photographic and schematic representations of the laser-heating system, viewed from above. Abbreviations of optical elements: objective (OBJ), achromat lens (AC), mirror (M), half-mirror (HM), mirror pinhole (PH), dichroic mirror (DM), beam splitter (BS), white light source (LI), phase-shifting plate (PP), notch filter (NF), longpass filter (LPF).

The system shares the basic characteristics with several stationary IR fiber laser on-axis LH systems, using the geoHEAT 60_NIR objectives in a compact arrangement (Fig. 2 ▸). For laser heating, it is compatible with most conventional DACs used by the community. For laser-heated X-ray spectroscopy near 90°, any DAC with a total radial opening angle of about 50° can be used. The initial design followed the one described by Boehler *et al.* (2009 ▸), which was optimized for XAS, with off-axis heating geometry and removable on-axis optical observation on both sides. However, on-axis alignment of the laser with respect to the DAC axis is beneficial for several reasons. We achieved this in a compact design by perforating the mirror upstream of the DAC with a 300 µm hole.

**Figure 3:**
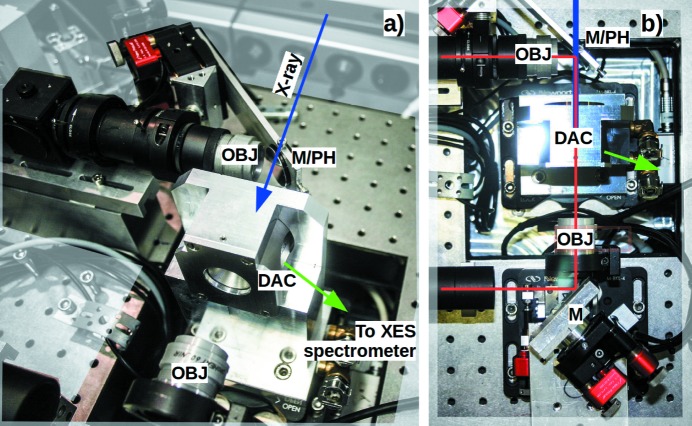
Close-up of the arrangement of the geoHEAT objectives (OBJ) and mirrors [(M), the right/upstream one with mirror pinhole (PH)] with respect to the DAC. (*a*) Isometric perspective. (*b*) View from above. Lines indicate the path of the laser and observation (red), incident X-ray (blue) and path of the salient X-ray emission to the spectrometer (green).

The beam of a single 100 W 1070 nm class 4 fiber laser (model YLR-AC100 from IPG Photonics) is split by a polarizing beamsplitter (BS; Thorlabs PBS25-1064-HP) into two separate beams to achieve double-sided heating. For independent tuning of the intensity of each side, each beam passes through a motorized λ/4 phase-shifting plate (PP) and a polarizing beamsplitter. The two laser beams are coupled coaxially with the optical observation paths (for both sides of the DAC) by dichroic mirrors (DM) (Fig. 2 ▸, right). The upstream mirror, installed at 45° with respect to both the objective and the DAC axis, has a pinhole of 300 µm diameter through which the X-ray beam passes without loss. The X-rays, laser path and optical path are thus coaxial with respect to each other (Fig. 3 ▸).

**Figure 4:**
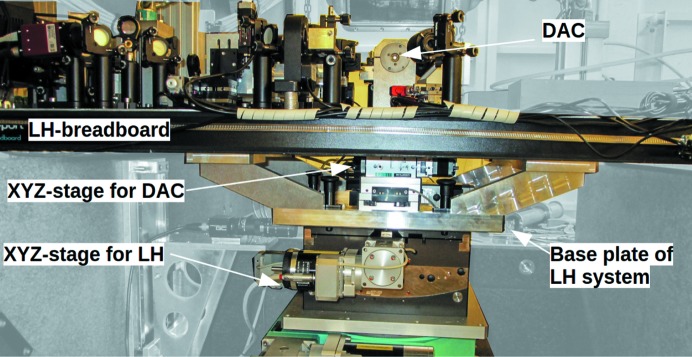
Side view of the laser-heating system. An aluminium construction, extending to 150 mm beneath the breadboard, hosts an *XYZ* translation stage for the DAC and serves as a basis plate to mount the entire system on a positioning stage at any beamline. See text for details.

The system is built on a 140 cm × 48 cm horizontal breadboard and weighs about 40 kg in total, excluding the laser and motor control unit. The sample, axis of optical observation and axis of the laser path are 105 mm above the breadboard (Fig. 4 ▸). An aluminium construction beneath the breadboard makes the breadboard mountable on an experimental end-station motorized stage, which enables alignment of the entire system with respect to the X-ray beam (Fig. 4 ▸). The aluminium construction beneath the breadboard also hosts a motorized *XYZ* stage aligning the DAC relative to the laser and observation optics. The system weight and dimensions make it transportable in the back of a car and mountable by two persons.

**Figure 5:**
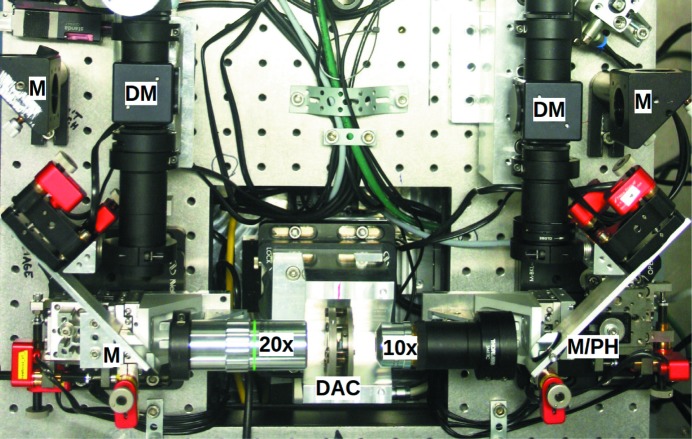
Close-up of the arrangement of the objectives of the laser heating system in laboratory mode. On the downstream side is a 20× Mitutoyo objective, on the upstream side a 10× Peleng objective, shown with a Boehler–Almax DAC in between (Boehler & De Hantsetters, 2004 ▸). Other optical elements are the same as in Figs. 2 ▸ and 3 ▸.

When the system is used as a stationary laboratory system, we exchange the geoHEAT 60_NIR objectives by Mitutoyo M Plan Apo 20× or similar objectives, and rearrange the setup in such a way that the objectives are inserted directly between the DAC and the 45°-mirrors (Fig. 5 ▸). This modification enables greater optical magnification and better imaging qualities. In this way, the system functions as a common stationary system.

### 2.2. Motorization and alignment   

For *in situ* laser-heated X-ray spectroscopy, the entire system rests on a beamline-owned translation stage at an experimental end-station. This stage enables alignment of the sample to the X-ray focus. An additional built-in *XYZ* translation stage beneath the DAC, controlled either by a 8SMC5 controller (Standa Ltd) or by beamline controllers, allows for positioning of the sample with respect to the laser focus. The DAC is first aligned with respect to the LH system, and then both are aligned together to the X-ray focus. The alignment can then be maintained remotely at any time.

The alignment of the X-ray focus with respect to the laser hot spot is achieved by centering both on the sample. The laser hot spot is centered visually, the sample is centered to transmission scans. In addition to this, in the case of XES, the incident X-ray beam causes sufficient optical fluorescence for visual alignment of the X-rays with respect to the sample.

A cooling circuit in the massive aluminium DAC holder (see plugs in Fig. 3 ▸, left) reduces thermal drift. *In situ* readjustment for each individual cell or condition is carried out with piezo motors (Newport GmbH), see Fig. 3 ▸. The same type of motors also enable stable observation of the sample on the mirror installed at the entrance of the spectrometer. Optionally, all motors can be incorporated into the beamline control system. The total number of motors is 15: 4 piezo motors for the laser fine alignment, 4 piezo motors for image positioning at the spectrometer entrance, 2 phase plate rotators, 3 axial DAC translations, and 2 motors for iris opening and closing.

### 2.3. Observation   

The major components enabling heating, imaging and reliable measurement of the temperature are two geoHEAT 60_NIR objectives from AdlOptica Optical Systems GmbH (OBJ in Fig. 3 ▸). They are optimized for laser light at 1070 nm and the observation wavelength range from 600 nm to 900 nm, and have a focal distance of 60 mm. The upstream objective is positioned perpendicular at 90° with respect to the DAC axis. The perforated silver mirror, installed at 45° between the geoHEAT objective and DAC-axis, aligns the optical path to the DAC-axis [Fig. 3 ▸(*a*)]. On the downstream side, the geoHEAT 60_NIR objective is positioned on-axis with respect to the DAC, such that the 45°-mirror is downstream of the objective [Fig. 3 ▸(*b*)]. No optical component needs to be moved in or out of any pathway for simultaneous heating, observation and X-ray measurements, which increases the overall stability.

Collimated by the geoHEAT objectives described above, the light for optical observation on each side is focused by achromatic doublets (AC) of 500 mm and 400 mm focal distance on the upstream and downstream side, respectively (AC254-500A and AC254-400A from Thorlabs), onto a silicon mirror with two pinholes (PH) of *ca* 50 µm diameter at the entrance of the spectrometer, as suggested by Boehler *et al.* (2009 ▸). The setup thus reaches magnifications of 8.3× and 6.7×.

**Figure 6:**
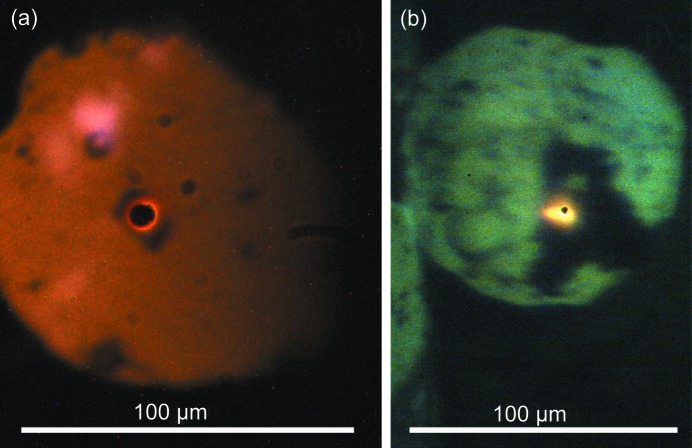
Images recorded during laser heating of a Pt foil at about 1900 K and 20 GPa. The clearly visible hotspot is positioned over the spectrometer pinhole for temperature measurement. Scale bars indicate image scale. (*a*) Heating with a geoHEAT 60_NIR objective at a magnification of 8.3×. (*b*) Heating with a Mitutoyo M Plan Apo 20× objective at a magnification of 25×. The diameter of the gasket hole is 100 µm in both cases. For comparison of magnification, the size of the spectrometer pinhole relative to the hot spot should be considered.

The upstream and downstream sides of the sample are imaged with a single camera (Manta 201C from Stemmer Imaging) observing the silicon mirror in front of the spectrometer. The sample images are aligned on the silicon mirror in such a way that the central parts of the upstream and downstream heated spots individually pass one of the two spectrometer pinholes. Fig. 6 ▸(*a*) shows an image of one side, recorded with a geoHEAT objective. The light transmitted through the holes is dispersed by a 300/500 nm grating of an Andor Shamrock spectrometer (163 mm focal distance) onto an Andor iDUS 420A image sensor.

When the system is used with Mitutoyo M Plan Apo 20× or similar objectives, the magnification increases to between 12× and 25×, see image of sample and hot spot in Fig. 6 ▸(*b*).

### 2.4. Temperature measurement   

The light from the two sides of the hot spot falls on separate regions on the image sensor resulting in two spectra. From these spectra, the temperature is calculated by the following steps. (i) The background is measured and later subtracted from all measurements. (ii) The spectrum is divided by the response of the optical system to a spectrum of known temperature from a tungsten filament lamp which was itself calibrated against a radiance standard from Gooch  Housego PLC. (iii) A Planck distribution is fitted to the spectrum. With this procedure, the melting temperature of a tungsten filament inside a halogen lamp at 3690 K is measured correctly to within 200 K. We use the program *T-rax* for continuous temperature measurements (Holtgrewe *et al.*, 2019 ▸). The acquisition time for temperature measurement is usually set to between 2 s and 4 s, but it can be freely varied. In addition, the light intensity can be dimmed by motorized irises on each side, located in the collimated path. The output power of the laser is stable, which allows for temperature stability in the hot spot over minutes and hours. We have measured laser-heated temperatures up to 3200 K. At very high heating temperatures, a longpass filter (LPF, FELH0500 from Thorlabs) can be inserted to suppress the UV light from the sample, which can reach the spectrometer detector via the second harmonics of the grating.

**Figure 7:**
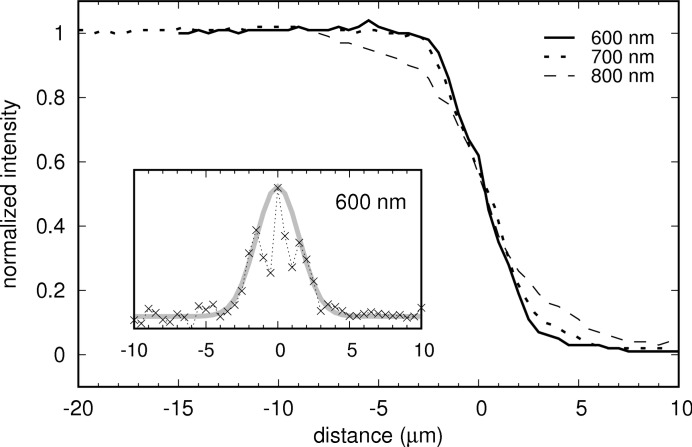
Spatial resolution of the optics and spectrometer, determined by sharp-edge scans at 600 nm, 700 nm and 800 nm. Inset: Gaussian fit (gray line) to the flanks of the gradient (crosses, shown for 600 nm) yields a FWHM of 4 µm.

The spatial resolution of the optics and spectrometer, important for reliable temperature measurements, has been determined for the geoHEAT objective at 600 nm, 700 nm and 800 nm by edge scans with narrow bandpass color filters. The scans are shown in Fig. 7 ▸. The shape of the derivative (the gradient) does not strictly follow a Gaussian distribution. This may be due to small inaccuracies in the shape of the pinhole at the entrance of the spectrometer or related to the light source. Nonetheless, we fit a Gaussian to both flanks to the gradient (shown for 600 nm in the inset of Fig. 7 ▸) in order to yield an estimate for the full width at half-maximum (FWHM) as an indicator of the spatial resolution. We obtained FWHM values of 4 µm, 5 µm and 6 µm at 600 nm, 700 nm and 800 nm at the full aperture of the objective. These values reflect the large working distance of the geoHEAT objective, but are in reasonable agreement with those of the recent careful study by Kantor *et al.* (2018 ▸).

**Figure 8:**
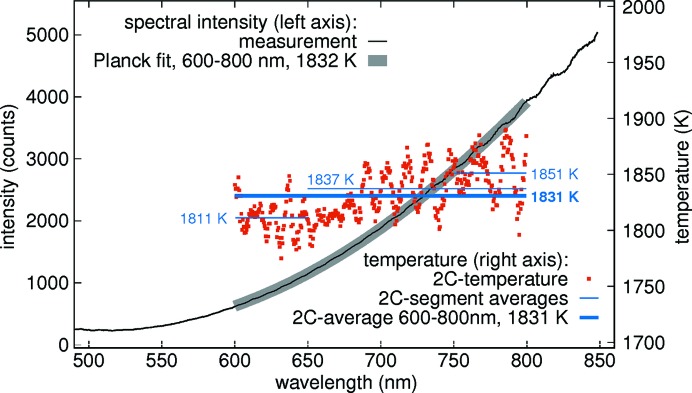
Temperature measurement on a Pt foil at about 30 GPa with a geoHEAT 60_NIR objective. A Planck fit (thick gray line, intensity scale on the left *y* axis) to the region of 600–800 nm of the recorded spectrum (black line, left *y* axis) yields 1832 K. The two-color (2C) temperatures (red dots, right *y* axis) and their averages over different wavelength regions reveal that the error in temperature due to chromatic aberration is 20 K. See text for discussion.

A spectrum of a laser-heated Pt foil at about 30 GPa, recorded with a geoHEAT 60_NIR objective, and a Planck fit to it over the wavelength range of 600–800 nm with a temperature of 1832 K (dark gray) is shown in Fig. 8 ▸ (spectral intensity on left *y* axis).

For assessment of the accuracy of the temperature measurement from inside the DAC, limited by chromatic aberration of the geoHEAT 60_NIR achromats and diamonds (Benedetti & Loubeyre, 2004 ▸; Benedetti *et al.*, 2007 ▸, 2009 ▸; Mezouar *et al.*, 2017 ▸; Kantor *et al.*, 2018 ▸; Giampaoli *et al.*, 2018 ▸), Fig. 8 ▸ also shows a so-called two-color plot of the same spectrum from a Pt foil inside the DAC (red dots, temperature axis on the right-hand side). The two-color plot shows a sequence of temperatures that result from many independent Wien function fits (Giampaoli *et al.*, 2018 ▸), each one to only two spectral intensities, the first at −25 nm relative to a given wavelength λ and second one at λ + 25 nm (Kantor *et al.*, 2018 ▸; Giampaoli *et al.*, 2018 ▸). In this way, chromatic aberration can be resolved, as it results in systematic temperature deviations between different regions of the same spectrum.

The red dots between 600 nm and 800 nm show short-range oscillations with a period of 20 nm that result from interference at one of the optical elements in the path. They are disregarded here. Instead, we consider averages of the two-color temperatures over wider regions, namely 600–650 nm, 650–800 nm, 750–800 nm and the complete 600–800 nm. They are shown as horizontal lines in the respective wavelength region, each one with the temperature indicated next to it (blue numbers in Fig. 8 ▸). To assess the magnitude of the error on temperature introduced by chromatic aberration, we first compare the 600–800 nm two-color average of 1831 K to the result of the Planck fit over the same region, 1832 K. This small deviation may be fortuitous. However, the average of the two-color temperatures in the central region of 650–750 nm is indistinguishable from the average of the region 600–800 nm, thereby indicating that the region 650–750 nm yields a robust average. The regions that deviate most from the 650–750 nm and 600–800 nm averages of 1831 K are 600–650 nm with an average of 1811 K and 750–800 nm with an average of 1851 K. Both roughly counterbalance each other, but the 600–650 nm region drags the total average of 600–800 nm stronger to lower temperatures.

We thus conclude that the error in temperature caused by chromatic aberration at about 1850 K is below 20 K and therefore minor, compared with the uncertainties caused by temperature- and wavelength-dependent emissivity (Jeanloz & Kavner, 1996 ▸), about which we have no information. We acknowledge that this source of error in the temperature estimation is present, but chose not to include it in the above uncertainty, which considers mainly the contribution from chromatic aberration.

## 3. X-ray spectroscopy techniques   

XES of iron-containing compounds allows determination of the electronic spin state of the iron. The spin state affects the structure, density and sound velocities of minerals in the Earth’s mantle. In oxides, the transition from the high-spin state under ambient conditions to the low-spin state in the lower mantle pressure range may cause an increase in the density of a mineral of up to 10% in the case of (Mg,Fe)O (Glazyrin *et al.*, 2017 ▸).

**Figure 9:**
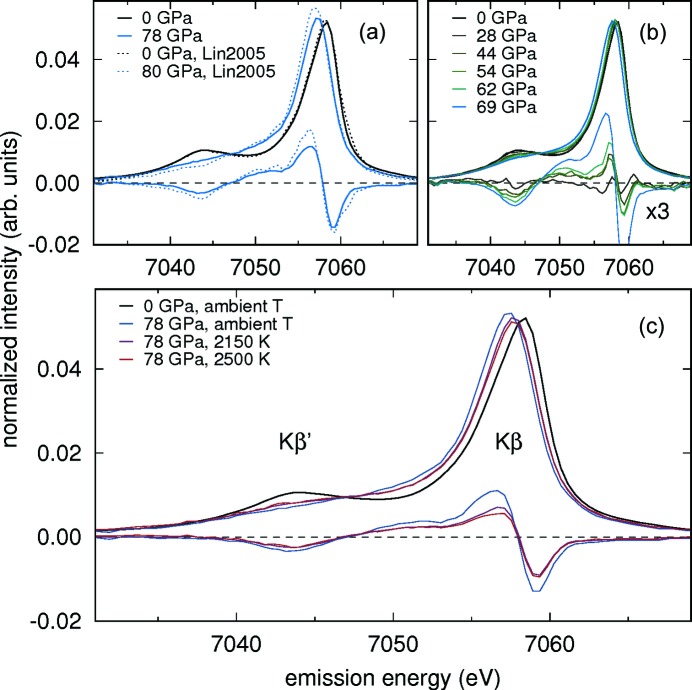
(*a*)–(*c*) XES spectra of Mg_0.67_Fe_0.33_O ferropericlase at high pressure and high temperature. The lower curves in each plot are difference curves with respect to the spectrum at the lowest pressure shown, ×3 in (*b*) for better visibility. Spectra are area-normalized and shifted to a common center of mass. (*a*) Comparison of ferropericlase spectra with those from Lin *et al.* (2005 ▸) at ambient and high pressure at ambient temperature. (*b*) Pressure sequence from ambient pressure to 69 GPa. (*c*) Temperature sequence at 78 GPa with differences with respect to ambient pressure. See Fig. 10 ▸ for evaluation.

The spin transition can be detected by changes in the *K*β emission line (3*p*–1*s* transition) which is split into the *K*β_1,3_ main line and the *K*β′ satellite [see Fig. 9(*c*) for the emission line labeling] due to 3*p*–3*d* exchange interactions (Badro *et al.*, 1999 ▸; Vankó *et al.*, 2006 ▸; Lin *et al.*, 2007 ▸).

For energy-dispersive XES measurements with a von Hámos spectrometer, the spectra are collected in a single-shot fashion, ensuring self-normalization. Measurements do not require motorized scanning of the emission signal. As there are no moving parts required in the dispersive geometry, energy-dispersive XES measurements have great potential for *in situ* iron spin transition measurements in the laser-heated DAC (Weis *et al.*, 2019 ▸). As described above, we used a panoramic cell in near-90° scattering geometry, passing the indentation wall of a common gasket (Weis *et al.*, 2019 ▸).

NIS is one of the few techniques to determine mean sound velocities of opaque materials, and compressional and shear wave sound velocities once the equation-of-state is known and the elastic line can be subtracted (Prescher *et al.*, 2015 ▸). When, due to opacity of the sample, Brillouin spectroscopy cannot be applied, and the sample environment is a laser-heated DAC, NIS is one of the methods of choice for fine powder materials (Zhao *et al.*, 2004 ▸; Kupenko *et al.*, 2015 ▸; Aprilis *et al.*, 2017 ▸).

## 4. Examples of application   

The LH system has been commissioned for XES and NIS at two different experimental end-stations of beamline P01, PETRA III, DESY, Hamburg (Wille *et al.*, 2010 ▸; Ketenoglu *et al.*, 2015 ▸).

### 4.1. X-ray emission spectroscopy of Mg_0.67_Fe_0.33_O ferropericlase   

We have recorded *K*β XES spectra of laser-heated Mg_0.67_Fe_0.33_O ferropericlase which was synthesized in a multi-anvil press and characterized and investigated in ealier work (Sinmyo *et al.*, 2014 ▸; Glazyrin *et al.*, 2017 ▸). Measurements were carried out using four 30 mm × 110 mm von Hámos crystals and a Pilatus 100K detector with a 20 min acquisition time for each spectrum. The X-rays were focused to 15 µm × 10 µm (H × V) on the sample.

Ferropericlase is a rock-forming mineral in Earth’s lower mantle. Its electronic spin state as a function of pressure, temperature and iron content continues to receive attention, as there is no consensus yet on the pressure and temperature region of the transition from high- to low-spin state (Lin *et al.*, 2005 ▸, 2007 ▸; Kantor *et al.*, 2006 ▸; Wentzcovitch *et al.*, 2009 ▸; Holmström & Stixrude, 2015 ▸; Glazyrin *et al.*, 2017 ▸).

We compressed a grain of Mg_0.67_Fe_0.33_O ferropericlase, loaded in NaCl as a pressure medium and thermal insulator, in several steps up to 80 GPa through the expected spin transition regime (Glazyrin *et al.*, 2017 ▸) and measured XES spectra.

In Fig. 9 ▸(*a*), we show the agreement of our spectra at ambient pressure and 78 GPa with the corresponding ones reported by Lin *et al.* (2005 ▸). Lower curves are the differences with respect to the ambient-pressure spectra. Fig. 9(*b*) shows the ambient-temperature sequence, from ambient pressure to 69 GPa. We laser heated the sample during decompression at 78 GPa, 71 GPa and 54 GPa. The laser-heated spectra at 78 GPa and up to 2500 K are shown in Fig. 9(*c*).

**Figure 10:**
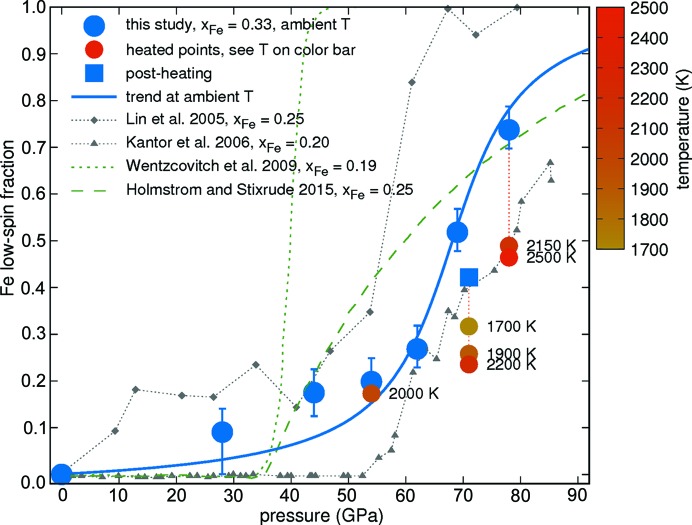
The iron low-spin component, computed from IAD of XES spectra (Vankó *et al.*, 2006 ▸) of ferropericlase as a function of pressure, compared with the literature data (Lin *et al.*, 2005 ▸; Kantor *et al.*, 2006 ▸; Wentzcovitch *et al.*, 2009 ▸; Holmström & Stixrude, 2015 ▸). Data taken at ambient temperature (blue circles) and laser heated (colored circles, see color bar and labels for temperature). The solid blue line represents the overall trend in our data. The iron content *x*_Fe_ of ferropericlase in the literature is indicated in the legend.

The iron low-spin-state component is calculated from the XES spectra by taking the normalized integrated absolute difference (IAD) between measured spectra and the ambient-pressure high-spin spectrum (Fig. 9 ▸) (Vankó *et al.*, 2006 ▸; Weis *et al.*, 2019 ▸). The resulting low-spin fraction shown in Fig. 10 ▸ was determined using the IAD between the spectrum of Lin *et al.* (2005 ▸) measured at 80 GPa (100% low spin) and our spectrum measured at 0 GPa (100% high spin). The error bars of 0.05 on the data points taken at ambient temperature (blue circles) reflect the measurement uncertainty and apply to all our data points in Fig. 10 ▸. At low pressures, noise in a spectrum results in overestimation of the IAD value, and therefore the error bar at 28 GPa to lower values is 0.1: the intensity of the *K*β′ line at 28 GPa is no less than at 0 GPa [see Fig. 9 ▸(*b*)]. This situation results in a fluctuation of the difference plot [black difference curve in Fig. 9 ▸(*b*), magnified by a factor of three for better visibility]. Such a difference deviates in overall shape as found for other differences characteristic for a spin state change and leads to an integrated absolute difference 0.1 higher than expected. We take account of this inherent overestimation in the presence of noise and uncertainty in data evaluation by indicating a larger error bar to lower values. We would like to note that not only changes in spin state, but to a smaller extent also pressure can affect the lineshape of the emission spectrum (Kantor *et al.*, 2006 ▸). Hence, using the spectrum at ambient pressure as a reference might result in an overestimation of the low-spin fraction [see offset of our results in comparison with Kantor *et al.* (2006 ▸) for pressures below 60 GPa].

Our ambient-temperature results (blue circles and blue solid arctan trend line) indicate that even at 78 GPa, our highest pressure, the transition had not occurred to 100% low-spin, but rather to 74%. The shift of the spin transition to higher pressures relative to the XES data reported by Lin *et al.* (2005 ▸) is not surprising as our sample has a higher iron content compared with 0.25 (Lin *et al.*, 2005 ▸). The spin transition pressure visible in our data is in good agreement with the compilation of spin transition pressures as a function of iron content published by Liu *et al.* (2014 ▸) and Glazyrin *et al.* (2016 ▸) considering error bars, while the results reported by Kantor *et al.* (2006 ▸) for ferropericlase with an iron content of 0.2 rather exceed the estimates of Liu *et al.* (2014 ▸). Literature data, from different measurement techniques and different iron contents of ferropericlase, show considerable scatter, which underlines the need for a greater number of measurements (Lin *et al.*, 2013 ▸; Liu *et al.*, 2014 ▸).

During heating at pressures where the iron is already partially in the low-spin state, *K*β′ increases in intensity. The evaluation in terms of low-spin fraction during laser heating (yellow to red circles in Fig. 10 ▸, see color bar for temperature) via the integrated absolute difference (IAD) method (Vankó *et al.*, 2006 ▸) reveals that the fraction of high-spin is increased, in agreement with the estimated spin state phase diagram, see *e.g.* Mao *et al.* (2011 ▸). We estimate the error of the IAD analysis of the high-temperature measurements to be 0.1, as compared with 0.05 of the ambient-temperature measurements. The error bars of the high-temperature measurements have been omitted for clarity.

Holmström & Stixrude (2015 ▸) estimated for Mg_0.75_Fe_0.25_O a low-spin fraction of about 20% at 2500 K and 78 GPa; Mao *et al.* (2011 ▸) measured for the same composition about 5% low-spin fraction at 2000 K and 80 GPa. Higher iron content shifts the transition to higher pressures and temperatures (Lin *et al.*, 2013 ▸). Our result of 50% low-spin fraction at 2500 K and 78 GPa is a reasonable value, in good agreement with the results of laser-heated XES reported by Lin *et al.* (2007 ▸). The laser-heated spectra at 71 GPa and 54 GPa consistently show a decrease in low-spin fraction compared with measurements at ambient temperature. The measurement at ambient temperature after laser heating at 71 GPa (blue square) reveals the reversability of the conversion to high spin during laser heating. The difference between values at 69 GPa (before laser heating) and 71 GPa (after laser heating) of about 0.1 confirms the magnitude of the summed uncertainty of data evaluation and sample annealing, as indicated by the error bars of the ambient-temperature data.

### 4.2. Nuclear inelastic scattering of FeSi   

We have recorded NIS spectra of the high-pressure phase of iron silicide FeSi (with B2 crystal structure) with the aim of determining the Debye velocity at high temperature. FeSi has been proposed as a potential candidate core material (Dobson *et al.*, 2002 ▸; Fischer *et al.*, 2014 ▸; Badro *et al.*, 2014 ▸; Tateno *et al.*, 2015 ▸). However, while the high-pressure sound velocities of the low-pressure phase FeSi in the B20 crystal structure have been determined at room temperature (Badro *et al.*, 2007 ▸), they are still experimentally unconstrained at elevated pressures and temperatures. Our sample material was synthesized at 23 GPa and ∼1750°C in an Al_2_O_3_ capsule in a multi-anvil press at the BGI in Bayreuth, Germany, and characterized using XRD and energy-dispersive X-ray fluorescence spectroscopy (EDS).

For laser-heated NIS measurements at beamline P01, a sample was loaded in a BGI-type panoramic cell with a beryllium gasket and KCl as a pressure medium and thermal insulator. The X-ray beam was focused down to 7 µm × 5 µm (H × V). The energy bandwidth of the high-resolution monochromator was 1.4 meV. Two avalanche photodiodes were inserted through the radial windows of the DAC at about 3 mm from the sample to collect the inelastic radiation scattered perpendicularly to the synchrotron X-ray beam. The energy dependence of the nuclear inelastic scattering was measured in the energy range −40 meV to 90 meV around the ^57^Fe nuclear resonance energy of 14.4 keV with an energy step of 0.2 meV. Nine spectra were collected over 40 min each and then summed to obtain the final spectrum. The time spectrum of nuclear forward scattering was recorded in the time domain at intervals between laser-heated NIS scans, with an APD moved into the path of the direct beam on the downstream side of the DAC.

**Figure 11:**
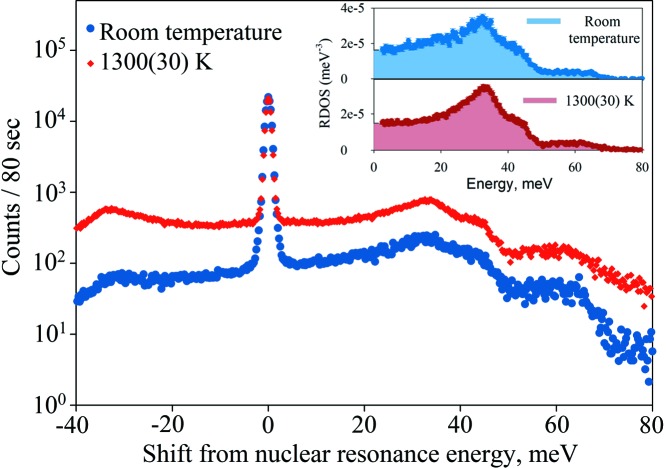
Energy dependence of nuclear inelastic scattering of iron silicide FeSi at 45 GPa, ambient temperature and 1300 K, collected at beamline P01, DESY. The peak at 0 corresponds to the resonance energy of the Mössbauer isotope. Collection time at 1300 K was 6 h. Inset: partial reduced phonon density of states (RDOS) of iron for FeSi at 45 GPa and indicated temperatures.

NIS spectra of FeSi, recorded at room temperature and 1300 K at 45 GPa, are shown in Fig. 11 ▸. The different transition probabilities at the Stokes and anti-Stokes sides between −40 meV and +40 meV have been used to infer the average temperature by assuming Bose–Einstein distribution of probabilities for phonon creation and phonon annihilation (Chumakov & Rüffer, 1998 ▸).

It has been shown that the bulk temperature of the laser-heated samples can differ from the temperatures measured on the surface of the sample by spectroradiometry due to axial and radial temperature gradients (Aprilis *et al.*, 2017 ▸; Campbell *et al.*, 2007 ▸). This difference can reach several hundred degrees for relatively thick samples employed for nuclear resonance scattering measurements (Kupenko *et al.*, 2015 ▸). Therefore, in these NIS measurements, we relied on spectroradiometry measurement only as a preliminary estimation during data collection. The actual temperatures of the samples were deduced from the intensity ratio between Stokes and anti-Stokes modes. The long-term stability of the heating during NIS scans is very good, similar to the one reported by Aprilis *et al.* (2017 ▸). After initial stabilization within tens of minutes, in some runs not a single further step of adjustment is necessary over the course of 8 h or more. In this time, several NIS scans (of about 1 h each) are carried out. Due to the poor statistics it is not possible to reliably estimate the temperature from the individual scans. However, summation of several consequent scans results in the same temperature within the error bars.

From the partial phonon density of states, we determined the mean sound velocity (Debye velocity) of the material and combined it with the elastic parameters from Fischer *et al.* (2014 ▸) in order to derive the longitudinal sound velocity (*V*_P_). We found that sound velocities in the B2-FeSi phase are higher than those in B20-FeSi at 45 GPa and ambient temperature [for B2-phase *V*_P_ = 9623 m s^−1^, for B20-phase *V*_P_ = 8912 m s^−1^ (Badro *et al.*, 2007 ▸)], and temperature also has a minor effect on the sound velocities of the B2-phase at pressures up to 45 GPa (*V*_P_ = 9764 m s^−1^ at 45 GPa and 1300 K). However, in order to fully constrain the temperature effect on the sound velocities, further experiments are required.

## 5. Comparison with other portable LH systems   

Despite compactness and portability, our system shares several important constructional elements of stationary LH systems installed at extreme conditions beamlines (Prakapenka *et al.*, 2008 ▸; Meng *et al.*, 2015 ▸; Anzellini *et al.*, 2018 ▸; Smith *et al.*, 2018 ▸), which make its use as easy as possible and temperature measurements as accurate as possible.

First to mention is the double-sided on-axis heating with specialized refractive objectives (in our case, geoHEAT 60_NIR). This offers a relatively easy and intuitive control and makes alignment of the laser straightforward. The upstream mirror is perforated, which enables zero attenuation of the incident beam while retaining full collinearity of the X-rays and laser beam. Full collinearity of the X-rays and laser is common to several stationary synchrotron LH systems, but rare, if not unique, in the domain of compact systems designed for X-ray spectroscopy (Boehler *et al.*, 2009 ▸; Dubrovinsky *et al.*, 2009 ▸; Kupenko *et al.*, 2012 ▸; Aprilis *et al.*, 2017 ▸; Kantor *et al.*, 2018 ▸; Giampaoli *et al.*, 2018 ▸).

Another important constructional aspect to mention is the double-sided radiospectrometric temperature measurement with two pinholes in the image plane at the entrance of the spectrometer. Such a scheme (Boehler & Chopelas, 1991 ▸) has been a common feature of stationary systems before the advent of more sophisticated 2D temperature measurement setups. The advantage lies in the full continuous optical control on which part of the hot spot on the sample is selected for temperature measurements. Another advantage is the low number of elements in the optical paths and the absence of fiber cables, which are present in other systems and make temperature measurement less straightforward.

The breadboard of the system makes it easy to mount and dismount an XRD detector and other additional detectors such as APDs or PIN-diodes. The potential of other constructional advantages have not yet been fully developed. For instance, the use of a single laser source can be a plus for future time-resolved measurements.

## 6. Conclusions and perspectives   

We have built and commissioned a portable LH system which is optimized for near-90° scattering angle spectroscopy techniques such as XES and NIS, while maintaining many important aspects of numerous stationary systems, namely high stability, full motorization, on-axis heating and observation, and optical pyrometry with only few optical elements. This portable system enables *in situ* X-ray spectroscopy at extreme pressure and temperature in a situation where *in situ* X-ray spectroscopy is a growing field but dedicated end-stations for X-ray spectroscopy at extreme conditions are scarce. Consequently, this portable LH system is the first with which XES spectra are reported. It is also the third system worldwide that enables laser-heated NIS, meeting strict requirements concerning signal-to-noise ratio by signal collection as close as possible to the sample at a ∼90° scattering angle.

Aside from its portability, notable features of this system are simultaneous on-axis heating and observation without attenuation of the incident beam by any part, achieved by a 300 µm hole in the upstream mirror, and direct alignment of the hot spot to the spectrometer entrance, resulting in accurate temperature measurements.

The combination of *in situ* X-ray spectroscopy with X-ray diffraction for an instantaneous control on sample structure is straightforward to implement and planned for the near future.

Laser heating and XES with a dispersive von Hámos spectrometer is a promising combination, as this single-shot spectroscopy setup allows for time-resolved XES measurements. In this way, powerful but less established measurement techniques such as valence-to-core XES (Glatzel & Bergmann, 2005 ▸; Weis *et al.*, 2019 ▸; Spiekermann *et al.*, 2019 ▸), HERFD-XANES and X-ray Raman scattering spectroscopy (Sternemann & Wilke, 2016 ▸; Weis *et al.*, 2019 ▸) can be combined with the sample environment of laser-heated DAC in the future.

As a future improvement, we will replace the Mitutoyo M Plan Apo 20× by Mitutoyo M Plan Apo NIR 20× objectives for a better optical performance over the entire visible range when used as a laboratory LH system. The focal spot of the laser will be enlarged.
